# Association of Body Mass Index with Chromosome Damage Levels and Lung Cancer Risk among Males

**DOI:** 10.1038/srep09458

**Published:** 2015-03-30

**Authors:** Xiaoliang Li, Yansen Bai, Suhan Wang, Samuel Mwangi Nyamathira, Xiao Zhang, Wangzhen Zhang, Tian Wang, Qifei Deng, Meian He, Xiaomin Zhang, Tangchun Wu, Huan Guo

**Affiliations:** 1 Department of Occupational and Environmental Health and Ministry of Education Key Lab for Environment and Health, School of Public Health, Tongji Medical College, Huazhong University of Science and Technology, Wuhan 430030, China; 2 Department of Occupational Disease, Institute of Industrial Health, Wuhan Iron & Steel (group) Corporation, Wuhan 430070, China

## Abstract

Epidemiological studies have shown an etiological link between body mass index (BMI) and cancer risk, but evidence supporting these observations is limited. This study aimed to investigate potential associations of BMI with chromosome damage levels and lung cancer risk. First, we recruited 1333 male workers from a coke-oven plant to examine their chromosome damage levels; and then, a cohort study of 12 052 males was used to investigate the association of BMI with lung cancer incidence. We further carried out a meta-analysis for BMI and male lung cancer risk based on cohort studies. We found that men workers with excess body weight (BMI ≥ 25 kg/m^2^) had lower levels of MN frequencies than men with normal-weight (BMI: 18.5–24.9). Our cohort study indicated that, the relative risk (RR) for men with BMI ≥ 25 to develop lung cancer was 35% lower than RR for normal-weight men. Further meta-analysis showed that, compared to normal-weight men, men with BMI ≥ 25 had decreased risk of lung cancer among both the East-Asians and others populations. These results indicate that men with excess body weight had significant decreased chromosome damage levels and lower risk of lung cancer than those with normal-weight. However, further biological researches were needed to validate these associations.

Lung cancer is one of the most common malignancies for males worldwide in terms of both incidence and mortality^1^. Cigarette smoking has been recognized as the major risk factors of lung cancer, but only a small number of smokers develop lung cancer, suggesting that some other factors such as air pollution, genetic susceptibility, or obesity may also play a role^2^,^3^.

Current emerging researches have recognized overweight and/or obesity as a significant risk factor for most common cancers^4^. One cohort study that had a mean follow-up of 5.4 years, showed that increased body mass index (BMI) is positively associated with increase on incidence of endometrial cancer, kidney cancer, and ovarian cancer^5^. It was established through meta-analyses that the risks of pancreatic cancer, gallbladder cancer, and liver cancer were significantly higher among over-weight and/or obesity individuals than individuals with normal-weight^6^,^7^,^8^. This phenomenon can be explained by the high level of insulin caused by the increased releases of free fatty acids, leptin, resistin, and TNF-α from adipose tissue, which can then promote cellular proliferation, inhibit apoptosis, and thus contribute to the carcinogenesis^4^. One study carried out in 125 Turkey individuals postulated that the over-weight/obese subjects had higher genomic damage levels than normal-weight individuals^9^. However, over-weight or obesity appears to have an inverse association with cancers strongly related to tobacco, in particular for lung cancer^10^. The above interpretations are difficult to explain this inverse association, and the mechanism linking BMI with lung cancer risk is largely unknown^11^.

Genomic instability coupled by chromosome damage is known to play important roles in initiation of lung cancer^12^. Environmental genotoxicants like polycyclic aromatic hydrocarbons (PAHs), which derived from smoking and occupational exposure, are thought to elicit lung cancer by increasing the extent of chromosome damage^13^. The cytokinesis-block micronucleus (CBMN) assay is one of the most well known method to evaluate the chromosome damage levels, while micronucleus (MN) frequency is a common biomarker for evaluating the risk factors of cancer^12^. The coke-oven workers represents a typical population of workers at high risk of getting lung cancer compared to the general population, because of the long-term occupational exposure to high PAHs contained in coke-oven emissions in their workplace^14^.

We thus, hypothesized that excess body-weight may affect individual's susceptibility to environmental genotoxicants and predisposition to cancer risk. To investigate this association of BMI with chromosome damage levels, 1333 male workers from a coke-oven plant were recruited for the study. First, we determined worker's exposure levels to carcinogenic PAH by measuring the plasma concentrations of benzo[a] pyrene-diolepoxide (BPDE)-albumin adducts, examined the chromosome damage levels by using the CBMN assay and measuring their lymphocytic MN frequencies. Furthermore, we conducted a prospective cohort study and a meta-analysis to investigate the association of BMI with lung cancer incidence among male populations.

## Results

### Cross-sectional study

#### Subjects characteristics

The general characteristics of 1333 study subjects are shown in Table 1. The levels of plasma BPDE-Alb adducts and lymphocytic MN frequencies in coke-oven workers were significantly higher than those of office-workers, (*P* < 0.001 and *P* = 0.005, respectively). However, there were no differences in BMI categories, percentage of smoking and alcohol drinking between coke-oven workers and office-workers. When compared with office-workers, coke-oven workers were generally a little younger (age: 42.22 ± 8.67 v.s. 43.45 ± 7.95; *P* = 0.016) and with less working years (21.10 ± 9.82 v.s. 22.32 ± 9.21; *P* = 0.037). The percentage of physical activity among coke-oven workers (47.1%) was also lower compared to office-workers (53.3%; *P* = 0.040).

#### Association of BMI with plasma BPDE-Alb adducts and lymphocytic MN frequencies

As shown in Table 2, among all subjects, men with excess body weight (BMI ≥ 25 kg/m^2^) had significant lower levels of plasma BPDE-Alb adducts (*P* = 0.023) and lower MN frequencies than men with normal-weight (BMI: 18.5–24.9) (FR = 0.89, 95%CI: 0.84–0.95) (Table 2). No such differences were seen for levels of plasma BPDE-Alb adducts and MN frequencies between under-weight (BMI < 18.5) and normal-weight men (*P* = 0.545, and 0.956, respectively).

Further stratified analysis showed that the FR (95%CI)s for men with BMI = 25.0–29.9 and BMI ≥ 30 were 0.89 (0.83–0.94) and 0.95 (0.80–1.13), respectively. After stratifying by workplaces, the associations of BMI ≥ 25 and BMI = 25.0–29.9 with decreased MN frequencies was seen among the two categories of workers, office-workers: [BMI ≥ 25: FR (95%CI) = 0.79 (0.70–0.89), BMI = 25.0–29.9: FR (95%CI) = 0.80 (0.71–0.91)], and the coke-oven workers [BMI ≥ 25: FR (95%CI) = 0.92 (0.86–0.99); BMI = 25.0–29.9: FR (95%CI) = 0.91 (0.85–0.99)] (Table 2). No significant association of BMI ≥ 25 with levels of plasma BPDE-Alb adducts among the office-workers (*P* = 0.608) was observed, but among the coke-oven workers, men with BMI ≥ 25 did have lower levels of plasma BPDE-Alb adducts when compared to men with normal-weight (*P* = 0.024).

Further stratification was done on all subjects based on age, years worked, smoking habit, alcohol drinking, and physical activity (Supplementary Table 1). In each of the three BMI strata, no significant difference in levels of MN frequencies was found between: smokers and non-smokers, between alcohol users and non-users, or between physically-active men and physically-inactive men (all *P* > 0.05). Among normal-weight subjects (BMI: 18.5–24.9), men with age >45 or working years >20 had significantly higher MN frequencies than men aged ≤ 45 or men who had worked ≤ 20 years, respectively (*P* = 0.019, 0.025). Furthermore, no significant interactions was observed between BMI and the above stratification variables (all *P* > 0.05) (Supplementary Table 1).

#### Cohort study

The baseline characteristics of the DFTJ cohort study subjects are shown in Table 3. Following an average of 4.5 years of follow up, a total of 208 men developed lung cancer, while 11 148 men did not develop lung cancer till end of 2013. The mean entry age of men with incident lung cancer was observed to be higher than the mean age of men who did not develop lung cancer till end of 2013 (69.1 years vs. 66.2years; *P* < 0.001). Compared men without incident lung cancers till 2013, men with incident lung cancer had more packing years of smoking (28.6 v.s. 18.0; *P* < 0.001). There was no difference in alcohol-drinking status between men with incident lung cancer and men without incident lung cancer (*P* = 0.119).

We then investigated the effect of BMI on the incidence of lung cancer among men. Table 4 shows that, when compared to the normal-weight men (BMI: 18.5–24.9), the RR (95%CI) for men with excess body weight (BMI ≥ 25) was 0.65 (0.49–0.88) (*P* = 0.005). Further analysis showed that the RR (95%CI) for men with BMI = 25.0–29.9 and BMI ≥ 30 were 0.65 (0.48–0.89) and 0.64 (0.30–1.37), respectively. In addition, a marginal increased risk of lung cancer was observed for under-weight men (BMI < 18.5) [RR (95%CI) = 1.80 (1.00–3.27), *P* = 0.051]. When we use BMI as a continuous variable in the Cox model, a significant inverse association was shown between BMI and incident lung cancer among men (*P*_trend_ = 0.005).

### Meta-analysis

#### Study search

A total of 2871 research articles from PubMed and Embase were obtained using the key words previously outlined. However, after applying the inclusion criteria previously described, only 22 research articles are selected which satisfy this criteria. Further critical evaluation of the selected 22 articles indicated the following: ten articles still did not meet the inclusion criteria, seven articles did not separately calculate RRs or HRs for men and women^15^,^16^,^17^,^18^,^19^,^20^,^21^, two article only divided BMI into two categories “Obesity ”(BMI ≥ 30) and “Non-Obesity”(BMI < 30)^22^,^23^, and one article did not have the result of RR or HR^24^. Thus, after evaluation of all the research papers, only 12 of the articles were included in this study meta-analysis having met all the inclusion criteria. The literature search and selection procedure are shown in Supplementary Figure 1.

#### Study characteristics

Supplementary Table 2 summarizes the general characteristics of the included studies. Combined, the 12 studies included 20793 incident cases with more than 33 million person years of follow-up. The mean follow-up years for these studies varied from 7.56 to 23.0. Among all studies: 4 studies examined East-Asian men^25^,^26^,^27^,^28^ and 8 studies examined other male populations^10^,^29^,^30^,^31^,^32^,^33^,^34^,^35^; 8 studies used measured body size data^27^,^28^,^29^,^30^,^31^,^32^,^33^,^34^ and 4 studies used self-reported body size data^10^,^25^,^26^,^35^; 4 studies used standard BMI categories based on WHO guidelines^10^,^32^,^34^,^35^, 1 study merged or classified the WHO BMI guidelines into new BMI categories^26^, 5 studies classified the BMI according to quartile or quintile of BMI cut-off points^25^,^29^,^30^,^31^,^33^, and 2 studies used the classifications of BMI proposed by WHO for the Western-Pacific region and adopted by previous studies on Asians^27^,^28^.

#### Association between BMI and risk of lung cancer

**Figure 1:**
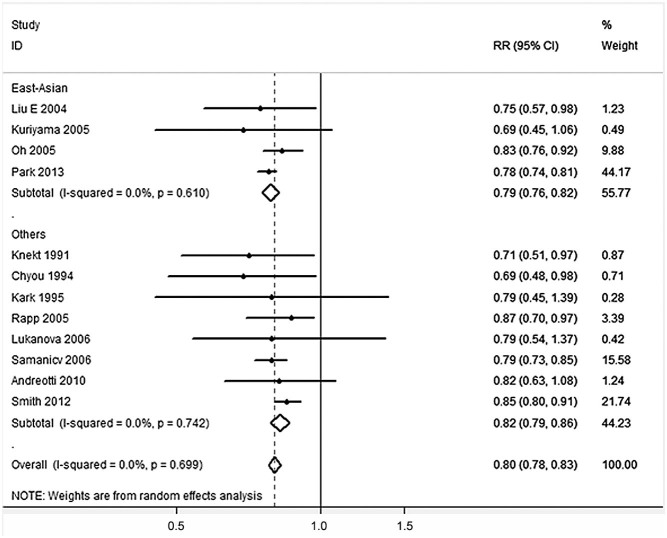
Forest plot for the association between BMI ≥ 25 and lung cancer risk.

As shown in Figure 1 and Table 5, overall analysis of all studies revealed that, when compared with normal-weight men, men with excess-body weight (BMI ≥ 25) had significantly lower risk of lung cancer [RR (95%CI) = 0.80 (0.78–0.83), *P* < 0.001], but under-weight men had a significantly increased risk of lung cancer [RR (95%CI) = 1.45 (1.35–1.56), *P* < 0.001]. Further stratified analysis showed that the RRs for men with BMI = 25.0–29.9 and BMI ≥ 30 were 0.86 (0.82–0.89) and 0.77 (0.74–0.80), respectively. After stratifying based on study population, the association of BMI ≥ 25 and BMI ≥ 30 with decreased risk of lung cancer was observed among both East-Asian men [BMI ≥ 25: RR (95%CI) = 0.79 (0.76–0.82); BMI ≥ 30: RR (95%CI) = 0.78 (0.75–0.82)] and other male populations [BMI ≥ 25: RR (95%) = 0.82 (0.79–0.86); BMI ≥ 30: RR (95%CI) = 0.69 (0.61–0.79)]. However, the association between under-weight and increased risk of lung cancer was observed only among East-Asian men [RR (95%CI) = 1.47 (1.36–1.58), *P* < 0.001], but not among other male populations [RR (95%CI) = 1.22 (0.90–1.66), *P* = 0.200]. Statistically heterogeneity was not observed in overall analysis and subgroup analyses (Table 5).

## Discussion

To our knowledge, this is the first study enrolling a large healthy occupational cohort whose findings indicated that men with excess body weight have significant low chromosomal damage levels when compared with normal-weight men. In this study, a new cohort study (DFTJ cohort) with 12 052 males was conducted in addition to meta-analysis of 12 previously published cohort studies to assess the associations between BMI and lung cancer incidence for male subjects. The research findings of the DFTJ cohort study showed that, comparison of normal-weight, men with BMI ≥ 25 had a decreased risk of lung cancer. Similar results were also observed after meta-analysis of the various studies. In addition, the cohort study findings, and meta-analysis also showed increased risk of lung cancer for Asian men with BMI < 18.5, when compared with normal-weight men.

PAHs are a group of environmental genotoxicants that are known to cause DNA damage and result in a dose-dependent risk of lung cancer^13^. When PAHs are absorbed by human body, they are metabolized by CYP enzyme where they finally form the ultimate carcinogen BPDE. BPDE then binds to the albumin, or the DNA to form BPDE-Alb or DNA adducts, which are important contributors to DNA damage^36^. Indeed, two previous studies had assessed the relationship between body size, fat content, and the levels of carcinogen-DNA adducts in white blood cells^37^,^38^. One study found that, after adjusting for some confounding factors, a significant inverse association of BMI with BPDE-DNA adducts in peripheral blood cell was found among 24 healthy cigarette smoking volunteers^37^. In the other study, Rundle et al recruited 143 healthy American, after a mean follow up of 12 moths, they found that BMI was inversely associated with the presence of detectable blood benzo[apyrene-DNA adducts^38^. These results suggested that an individuals' BMI and adipose content could have an important role in the metabolism of PAHs.

Measurement of MN frequencies in peripheral blood lymphocytes is frequently used in molecular epidemiology to evaluate the presence and the extent of chromosomal damage^39^. In this study, men with BMI > 25 had significant low levels of MN frequencies than normal-weight men among both coke-oven workers and office-workers. Some cross-sectional studies had reported an inverse association between BMI and oxidative DNA damage which is consistent with our findings^40^,^41^. For example, our previous study revealed a significant inverse association between increased BMI and urinary 8-hydroxydeoxyguanosine (8-OHdG), a biomarker of oxidative DNA damage^41^. Another earlier study from Denmark also found that weight loss was associated with increased levels of urinary 8-OHdG^40^. Since PAHs are known to be crucial to DNA damage, the findings that men with excess body weight may have lower levels of internal carcinogenic PAHs exposure, means the BMI-DNA damage association is thus biologically plausible.

Presently, very few published studies have demonstrated evidence that shows the association between body weight and lung cancer among Chinese subjects^42^. However, in 2010, Koh et al conducted a cohort study of 63 257 Chinese (both men and women) followed up between 1993 and 1998 in Singapore^21^. This research showed that the HR (95%CI) of lung cancer for participants with normal-weight (BMI: 20–23.9) and subjects with BMI < 20 was 1.22 (0.91–1.65) and 1.37 (1.00–1.88) respectively, when compared with subjects with BMI > 28 kg/m^2^^21^. In the DFTJ cohort study, we only analyzed male subjects and used BMI = 18.5–24.9 (WHO standard criteria for normal-weight) as the reference group. Compared to Koh's study, our cohort study was therefore better designed to investigate the relationships between BMI and incident of lung cancer among male Chinese.

In addition, we did meta-analysis that included 2.94 million subjects from 12 published cohort studies in order to analyze the association between BMI and risk of lung cancer. The results of this meta-analysis indicated that men with BMI ≥ 25 had a decreased risk of getting lung cancer than men with normal-weight among both the East-Asian and other populations. Indeed, one study by Renehan that did meta-regressions of study-specific incremental estimates, found a significant inverse association between a 5 kg/m^2^ increase in BMI and lung cancer incidence^11^. Another meta-analysis study that incorporated results from case-control and cohort studies found that overweight and obesity are protective factors against lung cancer in the general population for both genders^42^. Thus, although the findings of these studies are consistent with our findings as well, our study has unearthed new findings not previously known, notably that risk of lung cancer is high among under-weight males. However, meta-analysis of BMI and gallbladder, liver, and pancreatic cancer, showed that the risks of these cancers were significantly higher among over-weight and/or obesity individuals than normal-weight individuals^6^,^7^,^8^. The discrepancy effects of BMI on these digestive system cancers and lung cancer were probably due to the distinct mechanisms between these tumors. Obesity can result in gallstones, non-alcoholic fatty liver, and disorders of glucose, which, in turn, cause chronic inflammation and oxidative stress in these digestive organs and may further increase the cancer risks of these organs^4^.

The explanation for this observed inverse association between BMI and lung cancer is still not well known, although several theories have been advanced. Brennan reported that the rs9939609 A allele, for the obesity genetic marker *FTO* gene, which is linked with increased BMI, was associated with a decreased risk of lung cancer^43^. A recent genome-wide methylation analysis reported that increased BMI is associated with increased methylation at the *HIF3A* locus in blood cells and in adipose tissue^44^; this may possibly decrease the expression level of HIF-3α. Other studies have observed that HIF-3α can regulate many adaptive responses to hypoxia and expressions of numerous genes associated with angiogenesis, as well as cell survival and apoptosis^45^,^46^. These functions of the HIF-3α, thus suggests that it can potentially play a role in mediation of lung carcinogenesis. In this study, we found that men with BMI ≥ 25 had significant decreased levels of chromosome damage, which further supports the existing theories on possible mechanisms for this inverse association between over-weight subjects and lung cancer risk. However, the underline mechanisms linking BMI with different cancers still warrant further investigation.

The present study has some advantages. First, because there is a high gender difference in BMI and lung cancer etiology between women and men, we only carried out a men-specific investigation in both the occupational cohort and the meta-analysis. Second, all study included in our meta-analysis were cohort studies, which excluded the inherent limitations of case-control studies. However, the sample size for under-weight men in our occupational study was too small to evaluate the relationship between under-weight and chromosomal damage. Further biological and follow-up epidemiological studies with large sample sizes of population of interest were needed to validate and explore the possible mechanisms for the associations between BMI, DNA damage, and lung cancer risk observed in this study.

In conclusion, when compared with normal-weight men, men with excess body weight (BMI ≥ 25 kg/m^2^) had significant decreased levels of chromosome damage and lower risk of getting lung cancer, while East-Asian men with BMI < 18.5 had a significant increased risk of lung cancer. Further biological studies and large cohort studies were needed to validate these associations.

## Methods

### Cross-sectional study

#### Study population

The study subjects were recruited from a state-run coke-oven plant in Wuhan (Hubei, China). A total of 1333 male workers were selected in this study. Among them, 949 workers who had worked on the top, side, bottom, and adjacent workplaces of coke ovens at least for 1 year were referred as coke-oven workers, and 384 workers whose workplaces are offices are herein referred as office-workers. Once informed consent was obtained from the study participants, a standardized occupational questionnaire was used to collect the information on demographic characteristics such as; body weight, height, health status, smoking, alcohol drinking status, work history, and years worked.

Study participants were asked to give 5 mL of blood samples for analysis. 1-mL blood was used to conduct CBMN assay and the remaining was centrifuged to separate plasma from blood cells and stored at −80°C for other examinations. The subjects who had smoked more than one cigarette per day for at least one year were classified as smokers; otherwise, subjects were classified as non-smokers. The study was performed in accordance with relevant guidelines and regulations and approved by the Ethics and Human Subject Committee of Tongji Medical College (no. S320).

#### Measurement of BPDE-albumin adducts

The plasma BPDE-Alb adducts was measured using an ELISA method described by a previous study^41^. Briefly, 50 uL 0.1 M carbonate-bicarbonate buffer with 5 μg/mL rabbit antimouse IgG-Fc antibody (Jackson-immunoResearch, West Grove, PA) was added to 96 well-plates at 4°C and left overnight, and then each well was blocked with 15% non-fat dry milk (DFNM) dissolved in TBS-T. Then, 20 mL 3 μg/mL monoclonal antibody 8E11 (Trevigen, Gaithersburg, MD) was added to each well and incubated for another 1.5 h. After incubation, ABC reagent (Thermo scientific, Waltham, MA) prepared in TBS-T and tetramethylbenzidine was added to each well. Finally, 20 μl stop-buffer was added to stop the reaction and the colorimetric measurement was made at 450 nm using a micro plate spectrophotometer. The detection limit of the assay was approximately 1 ng BPDE-Alb adducts per microgram albumin, and the values below the detection limit were substituted with the values of 50% the detection limit for statistical analyses. Each standard or sample was prepared and analyzed in duplicate. The concentrations of plasma BPDE-Alb adducts were presented as ng/mg albumin.

#### Measurement of lymphocytic MN frequencies

We used a CBMN assay to measure lymphocytic MN frequencies and the detailed method has been described in our previous study^47^. In summary, approximately 0.5 mL of fresh whole blood is added to 4.5-mL RPMI-1640 medium supplemented (Gibco, Gaithersburg, MD) with 100 U/mL penicillin (Sigma-Aldrich, St. Louis, MO), 10% fetal bovine serum(Gibco), 2 mmol/L-glutamine (Sigma-Aldrich), and 1% phytohemagglutinin (Sigma-Aldrich), which is then incubated at 37°C and 5% CO_2_ for 44 hrs. Thereafter, cytochalasin-B (Sigma) at a concentration of 6 µg/mL is added to the culture medium, and incubated for an additional 28 hours. After incubation, the cells are fixed with 4:1 methanol/glacial acetic acid and put on clean slides. Finally, the cells are stained for approximately 13 min with 10% Giemsa solution (Sigma-Aldrich). Each sample is then examined microscopically and the number of binucleated cells containing MN were identified and recorded as MN frequency (‰). For quality control purposes, approximately 100 slides were randomly selected and blindly recorded by the researcher.

#### Cohort study

The design of the Dongfeng-Tongji cohort (DFTJ cohort) study has been described previously^48^. Briefly, a total of 27 009 retired employees living in the Shiyan City, including 12 052 men and 14 957 women, were enrolled in this cohort during the years 2008–2010. To establish a baseline for the study, after obtaining a written informed consent, researchers conducted a survey on all participants. The survey utilized a semi-structured questionnaire that probed participants health and demographic factors such as smoking history, medical history, height, weight, and other characteristics. After exclusion of male participants who had cancer prior to study follow up (n = 210), as well as those who had incomplete data such as; weight or height at baseline (n = 486), A total of 11 356 male subjects were finally included in this final analysis.

We then followed up these subjects to record their incidence of cancers. The type of the primary cancer and the date of cancer diagnosis were obtained from medical record. In this study, we mainly focused on the incidence of lung cancer and explored the association between BMI and lung cancer risk among male subjects. The study subjects were followed up until: (1) the end of year 2013, (2) or up to the day when a participant was diagnosed with (lung) cancer, (3) or until death, (4) or up to the point at which the subject was lost to follow up. The study was performed in accordance with relevant guidelines and regulations and approved by the Ethics and Human Subject Committee of Tongji Medical College (no. S335).

### Meta-analysis

#### Search strategy

We conducted a literature search in the PubMed (Medline) and Embase, between the years 1966 and 2013, with no language restrictions, for the association between BMI and risk of lung cancer. The key search terms utilized in this process were: “lung cancer”, “lung carcinoma”, and “lung neoplasm” in combination with “BMI”, “body mass index”, “obesity” and “body size”. In addition, we scrutinized references of retrieved literatures to identify further relevant studies.

#### Study selection

Research studies obtained were included in this meta-analysis if they satisfied the following criteria: (1) if the study design was cohort, (2) if the outcome was lung cancer incidence, (3) if description of under-weight/overweight/obesity was clearly defined in BMI (kg/m^2^), and (4) if the risk estimates included relative risks (RR) or hazard ratios (HR) with 95% confidence intervals (CI). Besides these criteria, studies that involved or focused on: non-human studies, conference abstracts, editorials, comment, and unpublished articles were excluded in the meta-analysis. Finally, if a cohort had been reported more than once, we used the most recent published study results.

#### Data extraction

Data was extracted and checked independently by two authors (XL and TW)). For each study, the following information was extracted: name of first author, year of publication, study location, participants' age and gender, duration of follow-up, number of participants incident cases, BMI categories, body size assessment method, lung cancer diagnostic method, and maximally adjusted risk estimates with 95% CIs for categories of BMI.

#### Data analysis

For the cross-sectional analysis and DFTJ cohort study, we divided the BMI into five categories as follow (BMI = weight in kilograms/height in meters^2^): BMI of 18.4 or lower, 18.5–24.9 (referent group), 25.0–29.9, 30.0 or more, and 25.0 or more. Based on the BMI categories as defined by the World Health Organization (WHO) for adults, we defined these categories as: “under-weight” (18.4 kg/m^2^ or lower), “normal-weight” (18.5–24.9 kg/m^2^), “excess body weight” (25.0 kg/m^2^ or more), “overweight” (25–29.9 kg/m^2^), and obese (≥30 kg/m^2^)^49^.

Between the coke-oven workers and office-workers, Pearson's χ2 test was used to compare categorical variables. Student's *t* test was used to compare mean value of age, years of employment, and BMI. Mann-Whitney *U* test was used for comparison of BPDE-Alb adducts, and univariate Poisson regression was used to compare the lymphocytic MN frequencies. Multiple Poisson regression models were used to analyze the associations between different BMI categories and MN frequencies, with adjustment for years of employment, smoking habit, alcohol drinking, and physical activity. We also conducted a subgroup analyses stratified by age, years of employment, smoking status, alcohol use, and physical activity among all subjects.

The Cox proportional hazards regression model was used to estimate RRs and 95% CIs of lung cancer incidence based on BMI categories when adjusted for potentially confounding variables. These analyses were done using the SPSS version 12.0 (SPSS Inc, Chicago, IL) software. Results were considered statistically significant when the two side *P* value < 0.05.

For studies used in the meta-analysis, the BMI cut-points in the original cohort studies were not all in accordance to the WHO criteria. To unify the classification, we used the WHO BMI cut-point values to represent the most approximate original BMI cut-points in these studies. During the meta-analysis, the summary RR estimates were calculated according to the different BMI categories and the method described by DerSimonian & Laird was used to combine the study-specific RR^50^.

The heterogeneity among studies was assessed using I^2^ statistic, which describes the proportion of total variation in point estimate that is due to heterogeneity. For the I^2^ metric, I^2^ values of 25%, 50%, and 75% were considered as cut-off points for low, moderate, and high degrees of heterogeneity, respectively. When heterogeneity was significant, we used a random effects model; otherwise, we used a fixed effect model. Forest plots were used to assess the overall risk estimate; and funnel plots were used to assess the overall publication bias. The meta-analyses were performed by using STATA 11.0 software (STATA Corp).

## Author Contributions

H.G. designed the experiment, X.L.L. and X.Z. executed the experiments, X.L.L. and Y.S.B. contributed to analyzing the data, X.L.L., S.H.W., S.M.N., Z.W.Z., T.W., Q.F.D., M.A.H., X.M.Z., T.C.W. and H.G. contributed to writing and editing the manuscript.

## Supplementary Material

Supplementary Information Supplementary Information

## Figures and Tables

**Table 1:** Distribution of general characteristics among male workers

Variables	Overall	Office-workers	Coke-oven workers	*P*
No. of subjects	1333	384	949	
Age (years, mean ± SD)	42.57 ± 8.48	43.45 ± 7.95	42.22 ± 8.67	0.016*
Length of work (years, mean ± SD)	21.46 ± 9.66	22.32 ± 9.21	21.10 ± 9.82	0.037*
Smoking status (n, %)				
Non-smokers	389 (29.2)	118 (30.7)	271 (28.6)	0.429†
Smokers	944 (70.8)	266 (69.3)	678 (71.4)	
Alcohol drinking (n, %)				
Non-drinkers	786 (59.0)	225 (58.6)	561 (59.1)	0.861†
Drinkers	547 (41.0)	159 (41.4)	388 (40.9)	
Physical activity (n, %)				
No	672 (51.1)	178 (46.7)	494 (52.9)	0.040†
Yes	642 (48.9)	203 (53.3)	439 (47.1)	
BMI [kg/m^2^, mean ± SD]	24.11 ± 3.04	24.20 ± 3.02	24.07 ± 3.06	0.491*
<18.5	27 (2.1)	9 (2.4)	18 (1.9)	0.879†
18.5~24.9	792 (60.2)	223 (58.8)	569 (60.7)	
25.0~29.9	456 (34.7)	134 (35.4)	322 (34.4)	
≥30.0	41 (3.1)	13 (3.4)	28 (3.0)	
≥25	497 (37.7)	147 (38.8)	350 (37.4)	
BPDE-Alb adducts [ng/mg albumin, median (25–75%)]	4.25 (3.63–5.05)	3.79 (3.13–4.40)	4.43 (3.85–5.32)	<0.001‡
MN frequency (‰, mean ± SD)	3.56 ± 2.66	3.19 ± 2.29	3.71 ± 2.79	0.005‡

*Student-t test.

^†^Two-sided χ2 test.

^‡^Mann-Whitney U test.

**Table 2:** Associations of BMI with plasma BPDE-Alb adducts and MN frequencies among male workers*

BMI (kg/m^2^)	n	BPDE-Alb	*P*†	MN frequency	FR(95%CI)	*P*‡
Overall						
18.5~24.9	777	4.30 (3.68–5.10)	---	3.70 ± 2.69	1	---
<18.5	27	4.52 (3.75–5.16)	0.545	3.70 ± 2.57	1.01(0.82–1.23)	0.956
25.0~29.9	448	4.18 (3.55–5.00)	0.041	3.27 ± 2.47	0.89(0.83–0.94)	<0.001
≥30.0	39	4.00 (3.17–4.82)	0.127	3.68 ± 3.07	0.95(0.80–1.13)	0.552
≥25.0	487	4.15 (3.51–4.95)	0.023	3.31 ± 2.52	0.89(0.84–0.95)	<0.001
P_trend_						<0.001
Office-workers						
18.5~24.9	222	3.79 (3.13–4.39)	---	3.50 ± 2.33	1	---
<18.5	9	4.40 (3.83–5.03)	0.068	4.12 ± 3.23	1.13(0.79–1.60)	0.512
25.0~29.9	132	3.78 (3.13–4.35)	0.724	2.84 ± 2.18	0.80(0.71–0.91)	0.001
≥30.0	13	3.76 (3.13–4.31)	0.443	2.38 ± 1.26	0.69(0.48–0.98)	0.035
≥25.0	145	3.78 (3.13–4.34)	0.608	2.80 ± 2.11	0.79(0.70–0.89)	<0.001
P_trend_						<0.001
Coke-oven workers						
18.5~24.9	555	4.49 (3.94–5.37)	---	3.88 ± 2.80	1	---
<18.5	18	4.63 (3.57–5.92)	0.983	3.72 ± 2.22	0.98(0.77–1.25)	0.882
25.0~29.9	316	4.31 (3.78–5.20)	0.034	3.51 ± 2.55	0.91(0.85–0.99)	0.014
≥30.0	26	4.41 (3.55–4.95)	0.269	4.08 ± 3.22	1.08(0.89–1.31)	0.535
≥25.0	342	4.33 (3.77–5.16)	0.024	3.55 ± 2.61	0.92(0.86–0.99)	0.030
*P*_trend_						0.054

*The values of plasma BPDE-Alb adducts were median (25%–75%), the values of lymphocyte MN frequency were mean ± SD.

^†^Mann-Whitney U test for comparison between different groups.

^‡^Multivariate Poisson regression with adjustment for: years worked, smoked habit, alcohol drinking, and physical activity.

**Table 3:** General characteristics for males in the DFTJ cohort

Variables	Incident lung cancer	Non-incident lung cancer	*P*
No. of subjects	208	11148	
Age (years, mean ± SD)	69.11 ± 6.63	66.16 ± 6.63	<0.001*
Pack-years (mean ± SD)	28.55 ± 25.35	18.00 ± 21.63	<0.001*
Smoking status (n, %)			
Non-smoker	46 (22.1)	4302 (38.6)	<0.001†
Smoker	162 (77.9)	6846 (61.4)	
Drinking use (n, %)			
Yes	116 (55.8)	5609 (50.3)	0.119†
No	92 (44.2)	5539 (49.7)	
Family history of tumor (n, %)			
Yes	1 (0.5)	315 (2.8)	0.042†
No	207 (99.5)	10833 (97.2)	
BMI [kg/m^2^, mean ± SD]	24.53 ± 3.35	24.10 ± 3.53	0.022*
<18.5	12 (5.8)	300 (2.7)	0.013†
18.5~24.9	128 (61.5)	5896 (52.9)	
25.0~29.9	61 (29.3)	4424 (39.7)	
≥30.0	7 (3.4)	528 (4.7)	
≥25	68 (32.7)	4952 (44.4)	

*Student-t test.

^†^Two-sided χ2 test.

**Table 4:** Association of BMI with risk of lung cancer among men in the DFTJ study cohort 2008–2013

BMI (kg/m^2^)	Incident cases	Person-years	RR (CI95%)	P*
18.5~24.9	128	23577.9	1.00	---
<18.5	12	1139.6	1.80 (1.00–3.27)	0.051
25.0~29.9	61	17782.8	0.65 (0.48–0.89)	0.006
≥30.0	7	2085.2	0.64 (0.30–1.37)	0.250
≥25	68	19803.7	0.65 (0.49–0.88)	0.005
*P*_trend_				0.005

Abbreviations: RR, relative risk.

*The Cox proportional hazards model was used, with adjustment for age, packing years, and family history of cancer when appropriate.

**Table 5:** Summary risk estimates of the association between BMI and lung cancer risk among male population

	Overall				East-Asians				Others			
BMI	N*	RR (95%CI)	*P*	*P*_het_†	N*	RR (95%CI)	*P*	*P*_het_†	N*	RR (95%CI)	*P*	*P*_het_†
18.5–24.9	12	1.00	---		4	1.00	---	1.00	8	1.00	---	1.00
<18.5	5	1.45 (1.35–1.56)	<0.001	0.075	3	1.47 (1.36–1.58)	<0.001	0.161	2	1.22 (0.90–1.66)	0.200	0.058
25.0~29.9	5	0.86 (0.82–0.89)	<0.001	0.281	0	NA	NA	NA	5	0.86 (0.82–0.89)	<0.001	0.281
≥30	7	0.77 (0.74–0.80)	<0.001	0.449	3	0.78 (0.75–0.82)	<0.001	0.989	4	0.69 (0.61–0.79)	<0.001	0.412
≥25	12	0.80 (0.78–0.83)	<0.001	0.699	4	0.79 (0.76–0.82)	<0.001	0.610	8	0.82 (0.79–0.86)	<0.001	0.742

*Number of the studies;

^†^*P* value for heterogeneity test.
