# Supplementary material for: Association of Body Mass Index with Chromosome Damage Levels and Lung Cancer Risk among Males
Source: Sci Rep. 2015 Mar 30;5:9458. doi: 10.1038/srep09458 (PMC4377580; doi:10.1038/srep09458)

Association of Body Mass Index with Chromosome Damage Levels and Lung Cancer Risk among Males

Xiaoliang Li [1], Yansen Bai [1], Suhan Wang [1], Samuel Mwangi Nyamathira [1], Xiao Zhang [1], Wangzhen Zhang [2], Tian Wang [1], Qifei Deng [1], Meian He [1], Xiaomin Zhang [1], Tangchun Wu [1], Huan Guo [1*]

[1] Department of Occupational and Environmental Health and Ministry of Education Key Lab for Environment and Health, School of Public Health, Tongji Medical College, Huazhong University of Science and Technology, Wuhan 430030, China;

[2] Department of Occupational Disease, Institute of Industrial Health, Wuhan Iron & Steel (group) Corporation, Wuhan 430070, China

***Corresponding Author:** Huan Guo, M.D., Ph.D., Institute of Occupational Medicine and Ministry of Education (MOE) Key Laboratory for Environment and Health, School of Public Health, Tongji Medical College, Huazhong University of Science and Technology, 13 Hangkong Rd, Wuhan 430030, Hubei, China Phone: 86-27-83657914; Fax: 86-27-83657765; E-mail: ghuan5011@hust.edu.cn

Table S1. Stratified analyses for the associations between BMI and MN frequencies among male workers

Variables	18.5≤BMI<25 kg/m^2			BMI<18.5 kg/m^2				BMI≥25.0 kg/m^2				$P_{interaction}$
	n	mean ± SD	FR(95%CI)	n	mean ± SD	FR(95%CI)	P [a]	n	mean ± SD	FR(95%CI)	P [a]	
Age												0.279
≤ 45	469	3.47 ± 2.43	1.00	15	2.93 ± 2.89	0.84(0.63-1.14)	0.270	295	3.10 ± 2.30	0.92(0.85-1.00)	0.041	
> 45	306	4.08 ± 3.00	1.00	12	4.67 ± 1.78	1.17(0.89-1.53)	0.662	190	3.59 ± 2.77	0.88(0.80-0.97)	0.007	
P^*		0.019			0.228					0.957		
Working years												0.138
≤ 20	383	3.35 ± 2.44	1.00	11	2.64 ± 2.91	0.79(0.54-1.14)	0.205	242	3.08 ± 2.25	0.93(0.85-1.02)	0.120	
> 20	394	4.07 ± 2.88	1.00	16	4.44 ± 2.10	1.10(0.87-1.40)	0.435	245	3.52 ± 2.71	0.87(0.80-0.94)	0.001	
P^*		0.025			0.966					0.190		
Smoking habit												0.230
Non-smokers	226	3.85 ± 3.04	1.00	6	4.67 ± 2.66	1.16(0.80-1.70)	0.432	144	3.22 ± 2.41	0.83(0.74-0.93)	0.002	
Smokers	551	3.67 ± 2.54	1.00	21	3.43 ± 2.54	0.96(0.76-1.22)	0.736	343	3.33 ± 2.54	0.92(0.85-0.99)	0.019	
P^*		0.326			0.546					0.682		
Alcohol drinking												0.684
Non-drinkers	441	3.56 ± 2.69	1.00	18	3.89 ± 2.74	1.07(0.84-1.36)	0.595	304	3.16 ± 2.44	0.89(0.82-0.96)	0.003	
Drinkers	336	3.93 ± 2.68	1.00	9	3.33 ± 2.29	0.87(0.60-1.25)	0.438	183	3.52 ± 2.58	0.90(0.82-0.99)	0.025	
P^*		0.337			0.202					0.182		
Physical activity												0.763
No	404	3.73 ± 2.49	1.00	13	3.92 ± 2.50	1.04(0.79-1.38)	0.780	246	3.25 ± 2.30	0.88(0.80-0.95)	0.002	
Yes	372	3.69 ± 2.90	1.00	14	3.50 ± 2.71	0.99(0.74-1.32)	0.960	240	3.35 ± 2.70	0.91(0.83-0.99)	0.032	
P^*		0.705			0.628					0.339		

[*] Multiple Poisson regression, adjusted for: years worked, smoking habit , alcohol use, and physical activity when appropriate.

Table S2. Characteristics of the 12 studies included in the meta-analysis

Authors	Publication year	Country	Age (years)	No of participants	Mean follow-up years	No of incident cases	Body size assessment	BMI categories	Diagnosis method
East-Asians									
Liu	2004	China	45-64	18249	13.57	467	self-reported at baseline	<19.5;19.5-24.5; >24.5	cancer registry
Kuriyama	2005	Japan	>40	12485	7.56	123	self-reported at baseline	18.5-24.9; 25.0-27.4; ≥27.5	cancer registry
Oh	2005	Korea	≥20	781283	10.29	2264	measured at baseline	<18.5;18.5-24.9;25.0-26.9; ≥27.0	pathology reports
Park	2013	Korea	30-80	1309144	10.5	10007	measured at baseline	<18.5;18.5-24.9; ≥25.0	cancer registry
Others									
Knekt	1991	Finland	20-75	25994	19	504	measured at baseline	<25; 25.1-27; >27	cancer registry
Chyou	1994	USA	not given	7840	23	236	measured at baseline	<26; ≥26.0	cancer registry
Kark	1995	Israel	40-69	9975	23	153	measured at baseline	<24.82; 24.82-28.31; ≥28.32	medical records
Rapp	2005	Austria	18-95	67447	9.6	464	measured at baseline	18.5-24.9; 25-29.9; ≥30.0	cancer registry
Lukanova	2006	Sweden	29-61	33424	8.2	59	measured at recruitment	18.5-25.3; 25.4-27.6; ≥27.7	cancer registry
Samanic	2006	Sweden	18-67	362552	19.1	162	measured at baseline	18.5-24.9; 25-29.99; ≥30.0	cancer registry
Andreotti	2010	USA	not given	39628	10	261	self-reported at baseline	<18.5;18.5-24.9; 25-29.9; ≥30.0	cancer registry
Smith	2012	USA	50-71	271238	9.7	6093	self-reported at baseline	<18.5;18.5-24.9; 25-29.9; ≥30.0	cancer registry

FIGURE LEGEND

Supplementary Figure 1. Flow diagram for search strategy and study selection for the meta-analysis.

Supplementary Fig.1

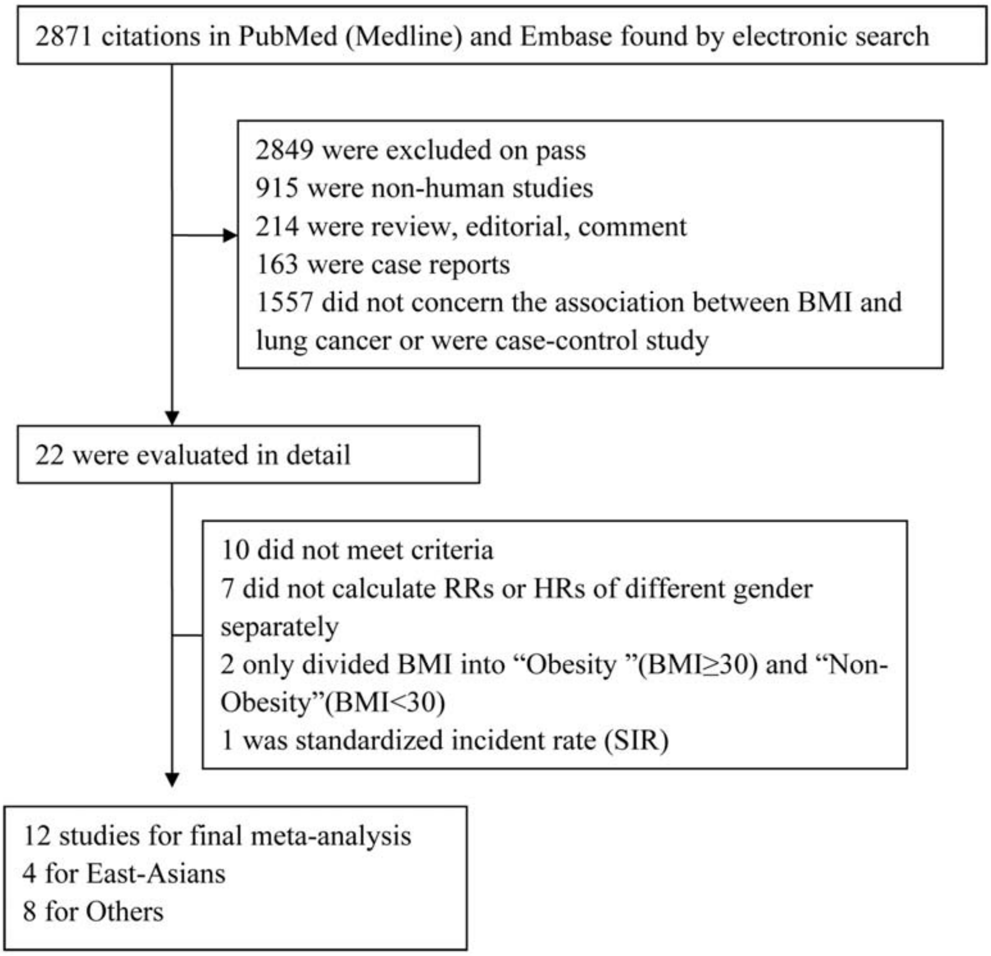

Supplement: Supplementary Information [file srep09458-s1.pdf]
